# Efficiency studies of modified IFAS-OSA system upgraded by an anoxic sludge holding tank

**DOI:** 10.1038/s41598-021-03556-6

**Published:** 2021-12-17

**Authors:** Mansour Fazelipour, Afshin Takdastan, Seyed Mehdi Borghei, Neda Kiasat, Marcin Glodniok, Paweł Zawartka

**Affiliations:** 1 Department of Environmental Engineering, Science and Research Branch, Islamic Azad University, Tehran, Iran; 2 Environmental Health Department, Ahvaz Jundishapur University of Medical Sciences, Ahvaz, Iran; 3 Department of Chemical and Petroleum Engineering, Sharif University of Technology, Tehran, Iran; 4 Department of Medical Mycology, School of Medicine, Ahvaz Jundishapur University of Medical Sciences, Ahvaz, Iran; 5 Department of Water Protection, Central Mining Institute, Katowice, Poland

**Keywords:** Biochemistry, Environmental sciences, Engineering

## Abstract

An upgraded integrated fixed-film activated sludge-oxic settling anoxic (IFAS-OSA) system is a new technology for reducing nutrients and excess sludge. The results showed that the average TN removal efficiency of the IFAS-OSA system was gradually increased up to 7.5%, while the PO_4_^–3^-P removal efficiency increased up-to 27%, compared with that of the IFAS system. The COD removal efficiency of the IFAS-OSA system was slightly increased up-to 5.4% and TSS removal efficiency increased up to 10.5% compared with the control system. Biomass yield coefficient (Y_obs_) in the IFAS and IFAS-OSA systems were 0.44 and 0.24 (gr MLSS/ gr COD). Hence, sludge production decreased by 45%. The average SVI was decreased by 48% in IFAS-OSA system compared with IFAS. This study demonstrated the better performance of the IFAS-OSA system compared to that of the IFAS system.

## Introduction

The nitrification and denitrification processes are of high importance in wastewater treatment plants because of the ammonia toxicity, oxygen demand, algae bloom and eutrophication in water bodies^14,16,17^. Insufficiently treated sewage are carrying plenty of nutrients and micropollutants that pose serious threats to receiving rivers^34^.

Another issue is the excess sludge which is generated from the biological treatment of wastewaters and must be disposed of in a safe and cost-effective manner for example in agricultural products^15,23^.

Reducing the nutrients and excess sludge in biological processes can be done by various techniques and methods. Some of these techniques consist of oxic-settling-anaerobic (OSA) process^18,28^, oxidation of the sludge by chlorine and ozone^5,11^. OSA technique is also a suitable solution for enhancement organic matter and nutrient (N & P) pollutants removal which significantly reduces biological excess sludge. This technology usually includes an aeration tank, a settling tank, and an anoxic/anaerobic tank in the return activated sludge line of the aerobic systems^1,33,35,37^.

Furthermore, some of the methods include the modified Ludzack-Ettinger (MLE) and integrated fixed-film activated sludge (IFAS)^6,26^. The IFAS technology increases the solid retention time without overloading the settling tank with solids and without the need to expand the aeration tank^4,39^. Combined with, OSA system enhances nitrification and denitrification and decreasing the excess sludge production and increases efficiency of IFAS process^20^.

Chudoba et al.^8^ compared the sludge yield of an OSA process with that of a CAS process and found that reduction of sludge yield from 0.48 to 0.13, the in the OSA system caused its reduction from 20 to 60%. The SVI was much lower and the ORP of -250 mV in the anaerobic tank showed a reduction of 36% in comparison with the ORP of + 100 mV. The OSA process had the excess sludge reduction of 58% compared with that of the CAS system^8^.

In Saby et al.^25^ also conducted similar research with CAS-OSA system. In his research ORP values in the anoxic and aerobic tanks were -250 mV and + 100 mV, respectively. The results revealed that the OSA process produced much less excess sludge than the control system^25^. Another researcher studied the OSA process for the reduction of biological sludge and found that the MLSS in a CAS-OSA process was reduced from 3000 mg/L to 2500 mg/L as the cellular mass production coefficient was reduced from 0.52 to 0.2 g biomass/g COD after 50 days with an ORP of -250 mV^10^.

In Vitanza et al.^29^ proved with his research on OSA technique in an anaerobic stage of the CAS system, that besides the good efficiency of the OSA system in the removal of COD, BOD, and nitrogen, the mass production coefficient (Y) was reduced from 0.6 to 0.4 g biomass/g COD. The ORP value was -160 mV while the COD, ammonia nitrogen, and phosphorus removal efficiencies were 76%, 82.5%, and 28%, respectively^29^. Also in research from Vitanza et al.^30^ observed the reduction of the excess sludge production in OSA technique of 49.6 ± 20.7% compared to the CAS system. Martins et al.^18^ research from 2020 has proven that better wastewater treatment performance was achieved using the OSA system (BOD_5_: 87%, TKN: 92%, NH^4^^+^–N: 94%) when compared with the CAS system (BOD_5_: 76%, TKN: 74%, NH^4^^+^–N: 78%), considering the organic matter and nitrogen removal rates.

In Corsino et. al.^9^. performed studies about the coupling of the OSA process with a thermal treatment at moderate temperature. The combination of the OSA process with the thermic treatment at moderate temperature (35 °C) enabled a very high efficiency of sludge minimization (80%), but lower nutrient reduction. Those research show a great potential for modification of oxic-settling-anaerobic technique.

The aim of this study was to investigate the performance of the new upgraded IFAS-OSA system in removing nitrogen, phosphorus, chemical oxygen demand (COD), and reducing excess sludge. The novelty of this study was the development of a new modified IFAS-OSA system for the first time by adding an anoxic sludge holding tank (ASHT) to the return sludge line of the system.

## Materials and methods

### Laboratory-scale IFAS, and IFAS-OSA systems

**Figure 1:**
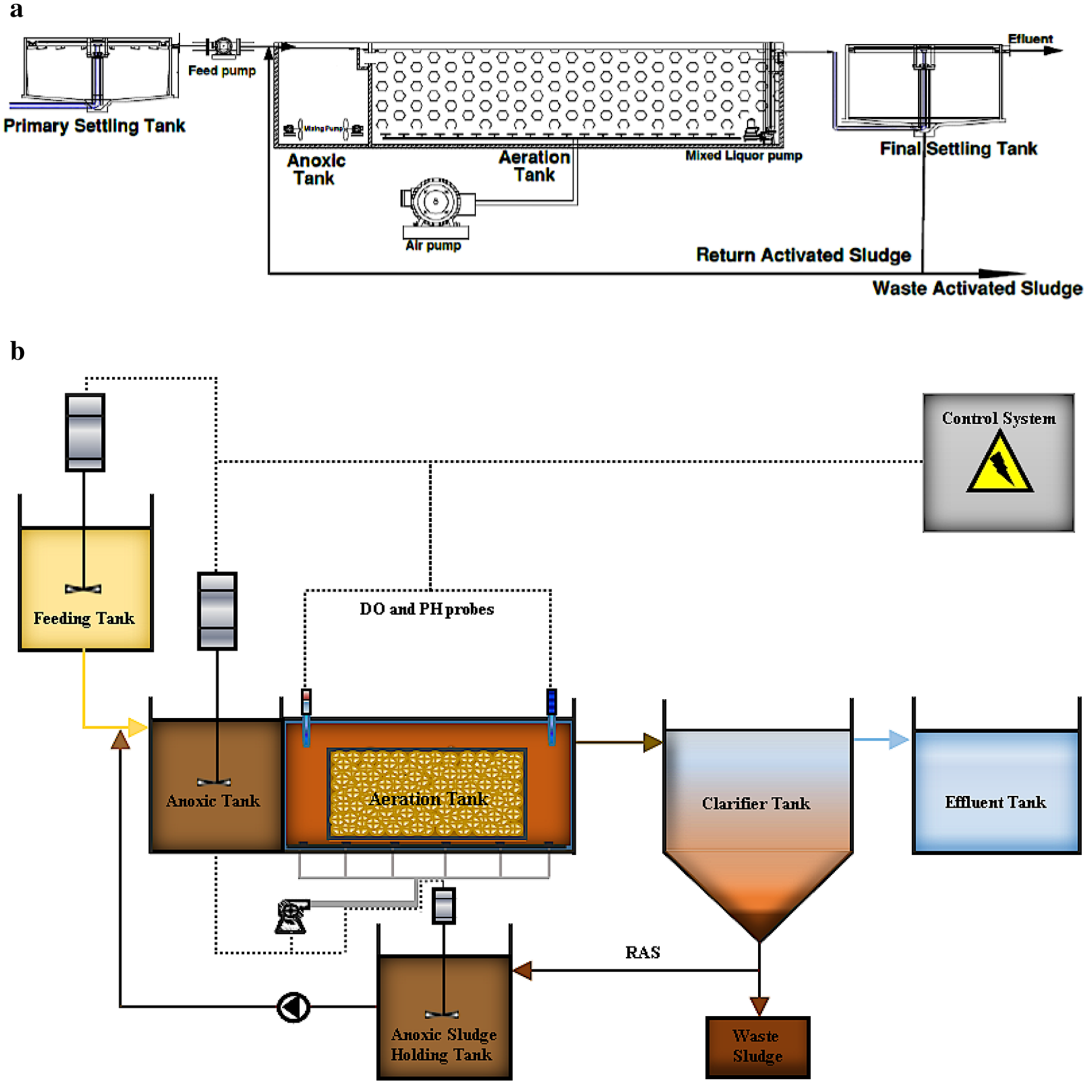
(**a**, **b**) The original plant setup and the experimental set-up; a: The original system, b: New upgraded IFAS-OSA system (created in Microsoft Word and Paint, link versions 2010&2013).

The IFAS reference system consisted of an automatic control system, a feeding tank, an equalization tank, a 72-L anoxic tank, a 144-L aeration tank, a sedimentation tank, an excess sludge tank, and an effluent tank. The modified IFAS-OSA system consisted of all the above-mentioned modules, however, it was extended with an additional anoxic sludge holding tank installed after excess sludge tank, before the effluent tank. In order to provide dissolved oxygen (4–6 mg/L DO), fine bubble diffusers were installed at the base of the aeration tanks. Aquarium pumps were used to achieve a complete mix in the bioreactors. In Fig. 1a,b schematic chart of the original treatment plant and the laboratory-scale IFAS-OSA system are presented.

### Media characteristics

The ${\mathrm{K}}_{3}$ media made of polyethylene with the bulk specific surface area of 584 m^2^/m^3^ and specific biofilm surface area of 325 m^2^/m^3^ at filling ratio of 50% were installed in the aerobic bioreactors in the fixed and attached operating mode.

### Wastewater characteristics

Urban wastewater was supplied from Choneybeh wastewater treatment plant and well homogenized in a feeding tank. The characteristics of the wastewater are shown in Table 1.

**Table 1:** The characteristics of the wastewater used in this study.

Constituent	Concentration
COD (mg/l)	325 ± 8.4
BOD (mg/l)	146 ± 5.4
TN (mg/l)	47 ± 3.2
TP (mg/l)	5.7 ± 1.05
PH	7.6 ± 0.076
Temperature (°C)	22 ± 2

### Start-up and operation of the bioreactors

To start up the IFAS and the modified IFAS-OSA bioreactors, the Qin (influent flows) of the systems were adjusted to 18 L/h in order to achieve 4 h HRTs in the aeration tanks, and 4 h HRT in the ASHT of the IFAS-OSA system. Then 1000 ml of the seed collected from the return activated sludge (RAS) line of Choneybeh wastewater treatment plant was added to the bioreactors. The seed sludge was mixed with wastewater being treated, so the process of biological decomposition takes place more rapidly. Simultaneously, the wastewater was injected into the systems. The bioreactors were operated in parallel mode for 60 days allowing the microorganisms to adapt, grow, and reproduce on the fixed media (K_3_) creating attached biofilms. A returned activated sludge (RAS) flow rate of 80% Q_in_ (Q_in_ is the influent flow rate) was applied to the IFAS and the upgraded IFAS-OSA system.

### The analytical methods

#### Physical–chemical analyses

After reaching steady state conditions in the IFAS and IFAS-OSA bioreactors, the main sampling and analyses were started. Chemical oxygen demand (COD) and total suspended solids (TSS) were measured according to the 5220B and 2540B methods, respectively, while the mixed liquor suspended solids (MLSS) was measured according to the 2540E method. Temperature and pH were measured by a pH meters (InoLab-Series WTW pH 720, Germany). DO was measured by a DO meters (Oxi 3210 SET 1, WTW, Germany). The total nitrogen and phosphorus were measured by a HACH DR5000 spectrophotometer (Merck, Germany) using the standard methods. All the above methods were extracted from standard methods for water and wastewater examination (APHA 2014). Total attached solid (TAS) was measured according to Kim et al.^17^.

### Microbial analyses

#### Fungal media preparations

The Sabouraud Dextrose Agar (SDA) media (Merck, Germany) was composed of dextrose (40 g/L), casein peptone (10 g/L), agar (15 g/L), distilled water (1000 mL), and chloramphenicol (0.05 mg/L). This media was used for isolation of filamentous *fungi* and yeasts. The differentiating media CHROMagar Candida (Paris, France) and Urease (Merck, Germany) were used for identification. All the media were prepared according to manufacturer ‘s procedure. Slide cultures were prepared for filamentous *fungi* and *yeasts*.

#### Isolation and identification of the *fungi*

During the experiment, fungal samples were gathered each day from the sampling sites. 1 ml of the mixed wastewater samples of each site (immediately after collection) was aseptically pipetted into 4 Sabouraud Dextrose Agar (SDA) media (Merck, Germany). Afterward, all the petri dishes were incubated at room temperature for 48–72 h and identification processes were performed on them. The average number of colonies (CFU/ml) was calculated. The fungal isolates were identified at the genus and/or species levels (36; 31).

#### Molecular analysis

The polymerase chain reaction-restriction fragment length polymorphism (PCR–RFLP) technique was employed to perform the molecular analysis. For the PCR amplification of the target sequences using a thermal cycler machine (Bio-Rad C1000, USA), the internal spacer region (ITS) of the yeast rRNA genes and two universal primers (including ITS1 [5'-TCCGTAGGTGAACCTGCGG-3'] and ITS4 [5' -TCCTCCGCTTATTGATATGC-3']) (Bioneer, South Korea) were utilized. Msp1 enzyme (Thermo Fisher Scientific, Waltham, MA, USA) was employed to digest the PCR amplicons. In order to evaluate the presence and length of the PCR amplicons and the profile of the digested products, electrophoresis was performed in a 1.8% agarose gel (Roche, Mannhiem, Germany) which was stained with SYBR green. Afterward, using ultraviolet transillumination, the gels were detected^19^.

### Isolation and identification of the *protozoa* and *metazoa* species

To isolate and identify the *protozoa* and *metazoa* species, the samples were collected once a week from the sampling sites. The identification (live observation) was carried out by an optical microscope (Olympus Microscopy, model: BH2, CH2) for a maximum of 3 h. All the *protozoa* and *metazoa* species were identified with an appropriate magnification. However, the staining method was used when necessary. The numbers of the observed *protozoa* and *metazoa* organisms were counted in three subsamples and then their average was calculated. For the identification of the *protozoa* and *metazoa* genera and species, several atlases and guides were used^2^.

### Nitrogen and phosphorus mass balances

Ammonia (NH_4_^+^–N) mass balances for anoxic tank:

1 $$ V\frac{{dN_{AX} }}{dt} = 0 = \left( {Q_{in} )\left( {N_{in} } \right) + (Q_{RAS} } \right)\left( {N_{R} } \right) - \left( {Q_{in} + Q_{RAS} } \right)\left( {N_{AX} } \right) $$

where $V\frac{{dN_{AX} }}{dt}$ is the ammonia (NH_4_^+^–N) rate in the anoxic tank. ${\text{Q}}_{{{\text{in}}}}$ is the influent flow. ${\text{N}}_{{{\text{in}}}}$ is the influent ammonia (NH_4_^+^–N) concentration. ${\text{Q}}_{{{\text{RAS}}}}$ is the returned activated sludge flow. ${\text{N}}_{{\text{R}}}$ is the ammonia (NH_4_^+^–N) concentration in the returned activated sludge flow. ${\text{N}}_{{{\text{AX}}}}$ is the ammonia (NH_4_^+^–N) concentration in the anoxic tank.

Ammonia (NH_4_^+^–N) mass balance for aeration tank:

2 $$ V\frac{{dN_{OX} }}{dt} = 0 = \left( {Q_{in} + Q_{RAS} } \right)\left( {N_{AX} } \right) - \left( {Q_{in} + Q_{RAS} } \right)\left( {N_{OX} } \right) $$

where $V\frac{{dN_{AX} }}{dt}$ is the ammonia (NH_4_^+^–N) rate in the aeration tank. ${\text{Q}}_{{{\text{in}}}}$ is the influent flow. ${\text{Q}}_{{{\text{RAS}}}}$ is the returned activated sludge flow. ${\text{N}}_{{\text{R}}}$ is the ammonia (NH_4_^+^–N) concentration in the returned activated sludge flow. ${\text{N}}_{{{\text{OX}}}}$ is the ammonia (NH_4_^+^–N) concentration in the aeration tank.

Nitrate (NO_3_–N) mass balances for anoxic tank:

3 $$ V\frac{{dNO3_{AX} }}{dt} = 0 = \left( {Q_{in} } \right)\left( {NO_{in} } \right) + \left( {Q_{RAS} } \right)\left( {NO_{R} } \right) - \left( {Q_{in} + Q_{RAS} } \right)\left( {NO_{AX} } \right) $$

where ${\text{V}}\frac{{{\text{dNO}}3_{{{\text{AX}}}} }}{{{\text{dt}}}}$ is the nitrate (NO_3_–N) rate in the anoxic tank. ${\text{Q}}_{{{\text{in}}}}$ is the influent flow. ${\text{Q}}_{{{\text{RAS}}}}$ is the returned activated sludge flow. ${\text{NO}}_{{{\text{in}}}}$ is the nitrate (NO_3_–N) concentration in the influent. ${\text{NO}}_{{\text{R}}}$ is the nitrate (NO_3_–N) concentration in the returned activated sludge flow. ${\text{NO}}_{{{\text{AX}}}}$ is the nitrate (NO_3_–N) concentration in the anoxic tank.

Nitrate (NO_3_–N) mass balances for aeration tank:

4 $$ V\left( {\frac{{dNO3_{OX} }}{dt}} \right) = 0 = \left( {Q_{in} + Q_{RAS} } \right)\left( {NO_{AX} } \right) - \left( {Q_{in} + Q_{RAS} } \right)\left( {NO_{OX} } \right) $$

where ${\text{V}}\frac{{{\text{dNO}}3_{{{\text{OX}}}} }}{{{\text{dt}}}}$ is the nitrate (NO_3_–N) rate in the aeration tank. ${\text{Q}}_{{{\text{in}}}}$ is the influent flow. ${\text{Q}}_{{{\text{RAS}}}}$ is the returned activated sludge flow. ${\text{NO}}_{{{\text{AX}}}}$ is the nitrate (NO_3_–N) concentration in the anoxic tank. ${\text{NO}}_{OX}$ is the nitrate (NO_3_–N) concentration in the aeration tank.

Phosphate (PO_4_^–3^–P) mass balances for anoxic tank:

5 $$ V\frac{{dPO4_{AX} }}{dt} = 0 = \left( {Q_{in} } \right)\left( {PO_{in} } \right) + \left( {Q_{RAS} } \right)\left( {PO_{R} } \right) - \left( {Q_{in} + Q_{RAS} } \right)\left( {PO_{AX} } \right) $$

where ${\text{V}}\frac{{{\text{dPO}}4_{{{\text{AX}}}} }}{{{\text{dt}}}}$ is the nitrate (PO_4_^–3^–P) rate in the anoxic tank. ${\text{Q}}_{{{\text{in}}}}$ is the influent flow. ${\text{Q}}_{{{\text{RAS}}}}$ is the returned activated sludge flow. ${\text{PO}}_{{{\text{in}}}}$ is the nitrate (PO_4_^–3^–P) concentration in the influent. ${\text{PO}}_{{\text{R}}}$ is the nitrate (PO_4_^–3^–P) concentration in the returned activated sludge flow. ${\text{PO}}_{{{\text{AX}}}}$ is the phosphate (PO_4_^–3^–P) concentration in the anoxic tank.

Phosphate (PO_4_^–3^–P) mass balances for aeration tank:

6 $$ V\left( {\frac{{dPO4_{OX} }}{dt}} \right) = 0 = \left( {Q_{in} + Q_{RAS} } \right)\left( {PO_{AX} } \right) - \left( {Q_{in} + Q_{RAS} } \right)\left( {PO_{OX} } \right) $$

where ${\text{V}}\frac{{{\text{dPO}}4_{{{\text{OX}}}} }}{{{\text{dt}}}}$ is the phosphate (PO_4_^–3^–P) rate in the aeration tank. ${\text{Q}}_{{{\text{in}}}}$ is the influent flow. ${\text{Q}}_{{{\text{RAS}}}}$ is the returned activated sludge flow. ${\text{PO}}_{{{\text{AX}}}}$ is the phosphate (PO_4_^–3^–P) concentration in the anoxic tank. ${\text{PO}}_{OX}$ is the phosphate (PO_4_^–3^–P) concentration in the aeration tank.

Determining the cellular mass production coefficient value (Y_obs_):

To determine the sludge production coefficient (Y_obs_), Eqs. 7 and 8 were used Fazelipour et al.^1^.

7 $$ {\text{dX}}/{\text{dt}} = {\text{YdS}}/{\text{dt}} $$

where dX/dt is the increase rate in the biomass concentration (MLSS) (mg/L) and dS/dt is the removal rate of the substrate (COD) (mg/L).

8 $$ {\text{Y}} = {\text{X}}_{0} {-}{\text{X}}/{\text{S}}_{0} {-}{\text{S}} $$

where S and S_0_ are the primary and ultimate substrate concentrations (mg/L), respectively. While X and X_0_ are the primary and ultimate biomass concentrations (mg/L), respectively.

It should be noted that temperature was maintained at 22 ± 2 ºC and the dissolved oxygen was kept in the range of 4 to 6 (mg/L) in the aeration tank.

The first-order kinetics for sludge decay without controlling the oxidation reduction potential in the ASHT were as follows (Eqs. 9 and 10):

9 $$ {\text{dX}}/{\text{dt}} = - {\text{bX}} $$

where X is the concentration of the biomass (mg/l) and b is the decay coefficient (d^−1^).

After integration, the sludge decay is expressed as:

10 $$ \Delta {\text{X}} = {\text{X}}_{{\text{t}}} - {\text{X}}_{0} = \left( {{1} - {\text{e}}^{{ - {\text{bt}}}} } \right) \cdot {\text{X}} $$

where ΔX is the decayed biomass (mg/l), X_t_ is the biomass concentration over time (mg/l), and X_0_ is the initial biomass concentration (mg/l)^32^.

## Results and discussion

After reaching steady state condition, the IFAS and IFAS-OSA systems were operated in parallel mode for 60 days and their results were compared. The results are presented in the following sections.

### Ammonia nitrogen (NH_4_^+^–N), NO_3_–N, TN, PO_4_^3–^P, COD, TSS, MLSS, and sludge volume index (SVI) and their removal efficiencies in the IFAS and IFAS-OSA systems

**Figure 2:**
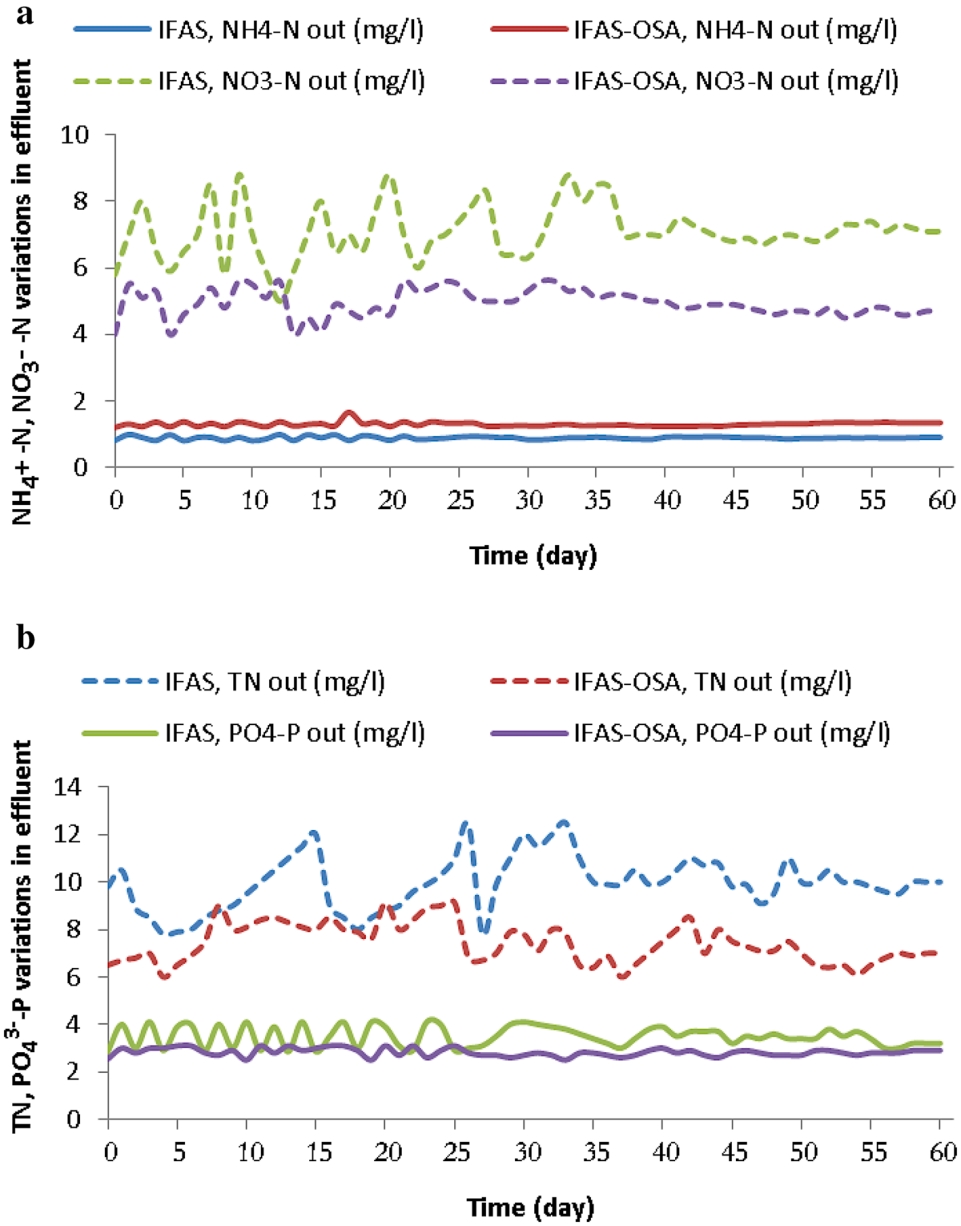
(**a**, **b**) Concentration variations of NH_4_^+^-N, NO_3_-N; b: TN, and PO_4_^–3^-P in the effluents of the IFAS and IFAS-OSA systems during the operation days.

The average steady-state concentrations and standard deviations of the NH_4_^+^–N measurements are listed in Table 2. Figure 2a shows the concentration variations of ammonia nitrogen (NH_4_^+^–N) in the studied processes. The observed removal efficiencies of NH_4_^+^–N are illustrated in Table 3. The results show that the average ammonia nitrogen (NH_4_^+^-N) were 98 ± 0.2%, 97 ± 0.3% for the IFAS and IFAS-OSA systems, respectively. Accordingly, the average ammonium (NH_4_^+^–N) removal efficiency slightly decreased in the IFAS-OSA system compared with the IFAS system. In 4 h retention time in the anoxic sludge holding tank (ASHT) the biomass went under starvation process. With the destruction of biomass in the ASHT, cell protoplasm containing ammonium ions was released into the return activated sludge. In the aeration tank some of these ammonium ions were observed by assimilation process, some were oxidized by the nitrifying bacteria (nitrification process), and the remaining part was poured into the effluent^7,27^. Table 4 shows the examples of ammonium nitrogen removal efficiency reported in previous and the current study^22,40^.

**Table 2:** The average steady-state concentrations and standard deviations of the NH_4_^+^–N, NO_3_–N, TN, PO_4_^–3^–P, COD and TSS measurements in the effluents of the IFAS and IFAS-OSA.

Processes	NO_3_–N	NH_4_–N	TN	PO_4_^–3^–P	COD	TSS
	Concentration (mg/L)	Concentration (mg/L)	Concentration (mg/L)	Concentration (mg/L)	Concentration (mg/L)	Concentration (mg/L)
IFAS	7.5 ± 1.5	0.9 ± 0.09	10.5 ± 2.5	4 ± 0.6	25 ± 4	16 ± 3
IFAS-OSA	5 ± 0.8	1.5 ± 0.08	7.5 ± 1.5	3 ± 0.4	10 ± 3	6 ± 1.5

**Table 3:** Nitrogen, phosphorus, TSS, and COD removal percentages in the effluents of the IFAS and IFAS-OSA system.

Processes	NH_4_^+^–N	TN	PO_4_^–3^–P	COD	TSS
	Removal efficiency (%)	Removal efficiency (%)	Removal efficiency (%)	Removal efficiency (%)	Removal efficiency (%)
IFAS	98 ± 0.2	80 ± 2.5	33 ± 8.2	92 ± 0.65	86 ± 3
IFAS-OSA	97 ± 0.3	86 ± 1.7	42 ± 3.6	97 ± 0.52	95 ± 1.5

**Table 4:** The examples of nitrogen and phosphorus removal efficiency (%) reported in previous and the current study.

Wastewaters	Process	Treatment				References
		Nitrogen removal efficiency		Phosphorus removal efficiency		
		Control	OSA	Control	OSA	
Urban wastewater	OSA + A	83^a^	77^a^	–	–	Zhou et al.^40^
Urban wastewater	MLE-OSA	70.23^a^	79.98^a^	31^b^	36-39^b^	Nikpour et al.^22^
Urban wastewater	IFAS-OSA	98^a^	97^a^	33^b^	40^b^	Current study

^a^NH_4_^+^–N removal.

^b^PO_4_^–3^ removal.

The average steady-state concentrations and standard deviations of the NO_3_–N measurements from the IFAS and IFAS-OSA are listed in Table 2. Figure 2a shows the concentration variations of nitrate (NO_3_–N) in the studied processes. As a result of adding ASHT to the return line of the upgraded system, the average NO_3_–N concentration in the effluent of IFAS-OSA system decreased to 5 ± 0.8 mg/L. Which indicate the increased denitrification process in the IFAS-OSA system^25^. According to Table 2 the average nitrate concentration decreased to 33% in the effluent of IFAS-OSA system compared with the IFAS system.

Total nitrogen (TN) measurements from the IFAS and IFAS-OSA are listed in Table 2. Figure 2b shows the concentration variations of TN in the studied processes. The observed removal efficiencies of TN are illustrated in Table 3. The results show that the average total nitrogen (TN) removal efficiencies were 80 ± 2.5%, 86 ± 1.7% for the IFAS and IFAS-OSA systems, respectively. By returning the carbonaceous biological oxygen demand resulted from anoxic degradation in the (ASHT) to the pre-anoxic tank, the BOD/TKN ratio and the TN removal efficiency increased as a result of biological denitrification process^38^. Table 3 shows that the TN removal efficiency in the IFAS-OSA system increased up to 7.5%, compared with the IFAS system.

The PO_4_^–3^–P measurements from the IFAS and IFAS-OSA are listed in Table 2. Figure 2b shows the concentration variations of phosphorus (PO_4_^–3^–P) in the studied processes. The observed removal efficiencies of PO_4_^–3^–P are illustrated in Table 3. The results show that the average phosphorus (PO_4_^–3^–P) removal efficiencies were 33 ± 8.2% and 42 ± 3.6% in the IFAS and IFAS-OSA systems, respectively. From Table 3, it can be concluded that the PO_4_^–3^–P removal efficiency of the IFAS-OSA system increased up-to 27%, compared with that of the IFAS system. This increase is the result of adding an ASHT to the RAS line of the upgraded IFAS-OSA system, which led to the energy transfer mechanism in the oxidation–reduction reactions (20). Also it was assumed that the phosphorus stored by the bacteria (PA _organisms_). Table 5 compare present work with other biological treatments.

**Table 5:** The examples of nitrogen and phosphorus removal efficiency reported in the present and previous studies.

Wastewaters	Process	Treatment				References
		Nitrogen removal efficiency (%)		Phosphorus removal efficiency (%)		
		Control	OSA	Control	OSA	
Synthetic Wastewater	SBR-OSA	–	–	84^d^	98^d^	Goel and Noguera (2006)
Synthetic Wastewater	MBR-OSA	100f.	100^e^	95^d^	90^d^	Datta (2009)
Synthetic Wastewater	*CAS-OSA*	54^a^	62^a^	67^b^	79^d^	Troiani (2011)
Synthetic Wastewater	*CAS-OSA*	49^a^	58^a^	28^b^	30^d^	Ye (2008)
Urban Wastewater	IFAS-OSA	98^c^	97^c^	33^b^	42^d^	This study

a: TN removal, b: TP removal, c: NH_4_ removal, d: PO_4_^–3^ removal, e: NH_3_ removal.

**Figure 3:**
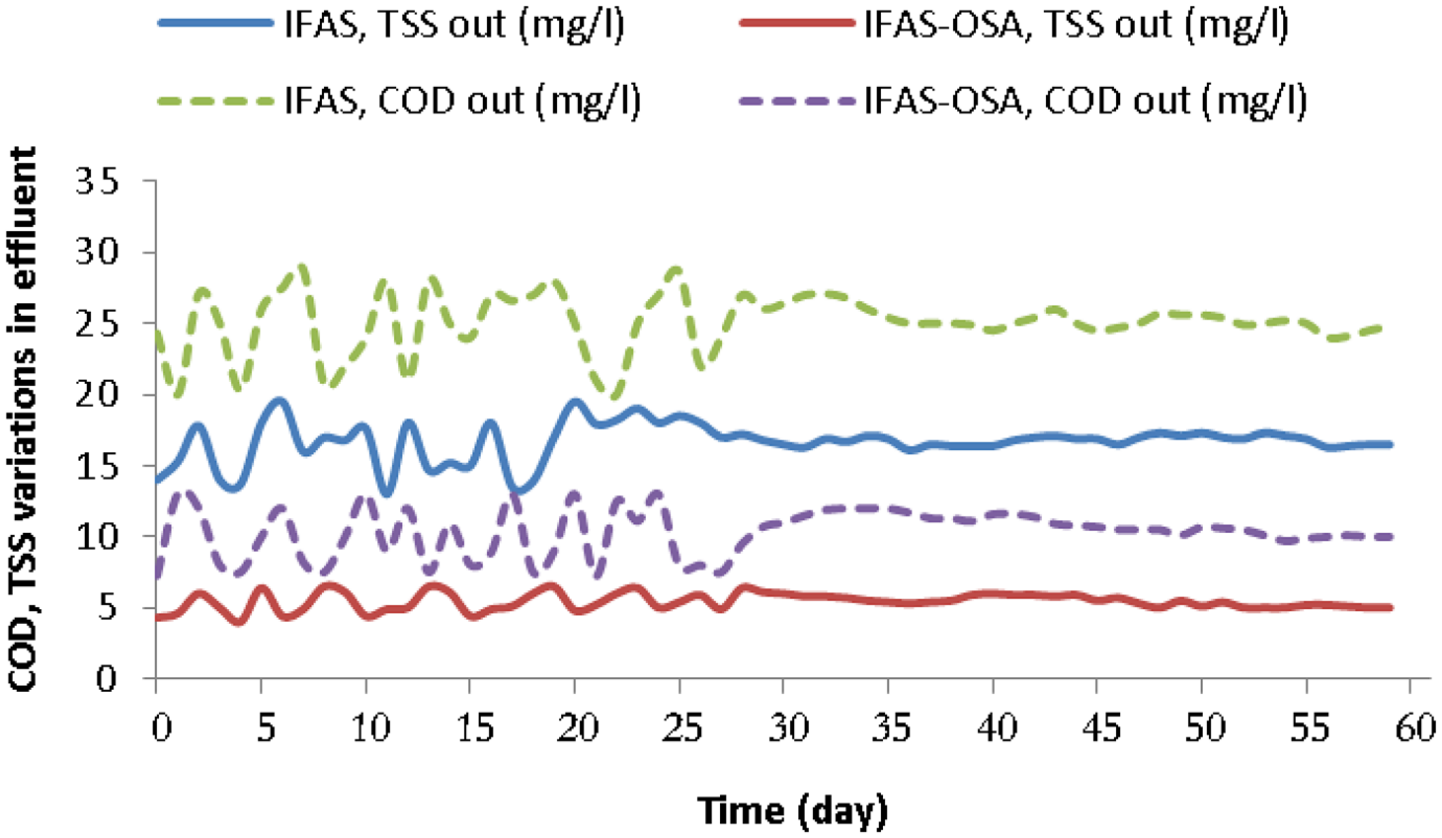
Concentration variations of TSS and COD of the IFAS and IFAS-OSA systems during the operation days.

The COD and TSS measurements from the IFAS and IFAS-OSA are listed in Table 2. Figure 3 shows the concentration variations of COD and TSS in the IFAS and IFAS-OSA systems. The observed removal efficiencies of COD and TSS are illustrated in Table 3. The results show that the average COD and TSS removal efficiencies in the FAS and IFAS-OSA systems were 92 ± 0.65%, 97 ± 0.52% and 86 ± 3%, 95 ± 1.5%, respectively. The average TSS in the effluent of the IFAS-OSA system was 6 mg/L, Therefor the TSS concentration decreased by 62.5%, and the TSS removal efficiency of the IFAS-OSA system was increased up to 10.5% compared with the control system. This showed the positive effect of adding the ASHT to the return sludge line of the IFAS system^22^.

According to Table 2, the average COD concentration in the effluent of the IFAS-OSA system was 10 mg/L, so the COD concentration decreased by 60%, compared with the IFAS system. With the SRT of 10 days and HRT of 4 h (Table 6), the COD removal efficiency of the IFAS-OSA system was slightly increased up-to 5.4% compared with the control system. Therefore, this increase could be duo to the ASHT (longer HRT). Saby et al. in 2003 also reported that the OSA system was able to increase the COD removal efficiency (25). But in 2008, Ye et al. reported that the OSA system decreased the COD removal efficiency in HRTs of 5.5 and 7.6 h^39^.

**Table 6:** The comparison of the operational parameters, ORP levels, sludge production (Y_obs_), and excess sludge reduction of the IFAS and IFAS-OSA processes in the current study.

Study by		Saby	Fazelipour	
Processes		MBR-OSA	IFAS	IFAS-OSA
Parameter	Unit			
OLR	g COD/ gMLSS.d^-1^	0.66	0.35	0.38
MLSS	mg/L	2000	3155	2461
Att. B	g/m^2^	-	22	26
SVI	ml/g	100	96	50
TSS_EFF_	ml/g	17	16	6
SRT	Day	19	10	10
HRT	H	6	4	4
ORP	mV	+ 100∼ -250	+ 85	− 148
Y_obs_	g MLSS/ g COD	0.32∼ 0.18	0.44	0.24
Q_Excess_	g/d	47∼23	45	35
ESR	%	36	Control	22

OLR: Organic loading rate; MLSS: Mixed liquor suspended solid; Att. B: Attached Biomass; SVI: Sludge volume index; TSS _Eff_: Total suspended solids in the effluent; SRT: Sludge retention time; HRT: Hydraulic retention time; Q_in_: Influent flow; Q_RAS_: Returned activated sludge flow; ORP: Oxidation reduction potential; Y_obs_: Observed yield coefficient; S_Excess_: Excess sludge; ESR: Excess sludge reduction.

The average concentrations of MLSS and SVI measurements from the IFAS and IFAS-OSA systems are presented in Table 6. According to Table 6 the average MLSS and SVI concentrations were 3155 (mg/l), 2461 (mg/l) and 96 (ml/g), 50 (ml/g) for the IFAS and IFAS-OSA systems, respectively. The average MLSS and SVI concentrations in the IFAS-OSA system decreased by 22% and 48%, respectively compared to that of IFAS system. Also the sludge was more cohesive and the sludge settleability was improved. In 2010, Kim et al. reported that this may be due to the intracellular polymers (bridging mechanism) under anoxic conditions^17^.

### Effects of the ASHT on oxidation–reduction potential (ORP), Observed yield coefficient (Y_obs_), and Excess sludge flow rate (Q_Excess_) in the IFAS and IFAS-OSA systems

**Figure 4:**
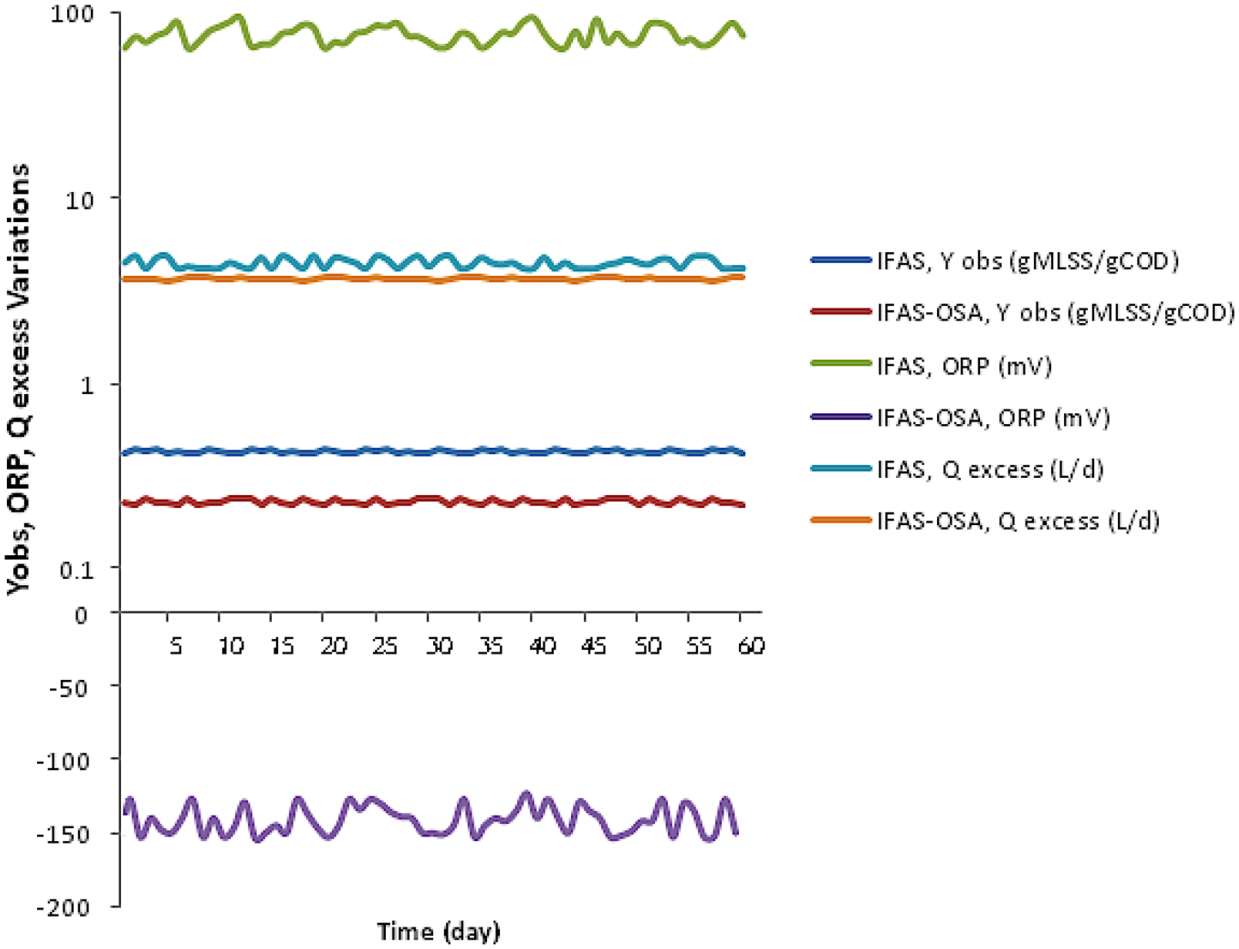
Variations of Y_obs_, ORP and Q_Excess_ in the effluents of the IFAS and IFAS-OSA systems during the operation days.

Figure 4 shows the oxidation reduction potential (ORP) variations after reaching steady-state conditions in the IFAS and IFAS-OSA systems during the operation days. Table 6 shows the average ORP levels for the IFAS and IFAS-OSA systems in the aerobic tank, and the anoxic sludge holding tank (ASHT). The average ORP values of the IFAS and IFAS-OSA systems were + 85 ± 16 and -148 ± 14 mV. In the ASHT the ORP levels gradually decreased during the operation days. These results are consistent with the results of Wang et al. study. As the oxidant (NO_3_–N) decreased and the substrate was released into the solution of anoxic sludge holding tank, the ORP levels decreased gradually in the retention times of 4 h. However, due to the limited retention time in the sludge holding tank, the ORP level could not drop to—250 mV^32^.

Figure 4 and Table 6 show the observed yield coefficient (Y_obs_) variations and the average values (Y_obs_) of the IFAS and IFAS-OSA processes in the bioreactors. Biomass yield coefficient (Y_obs_) in the IFAS and IFAS-OSA systems were 0.44 and 0.24 (gr MLSS/ gr COD), respectively. Therefore, the sludge production of the IFAS-OSA system decreased by 45%, compared to that of the IFAS system. This result is in agreement with those of the study done by Demir et al. and Vitanza et al. In their studies the biomass yield coefficient (Y_obs_) was reduced from 0.48 to 0.13 in the OSA process, while the sludge production was reduced from 20 to 60%[10; 29].

Figure 4 and Table 6 show the daily excess sludge variations and the average excess sludge flow rate (Q_Excess)_ of the IFAS and IFAS-OSA systems during operation. The average excess sludge (Q_Excess)_ values in the IFAS and IFAS-OSA systems were 4.5 ± 0.6 and 3.5 ± 0.4 (L/d), respectively. The data also showed that the excess sludge of the IFAS and IFAS-OSA system with SRT of 10 days and HRT of 4 h were 45 and 35 (gr/day), respectively. According to Table 6, the daily Q_Excess_ sludge in the IFAS-OSA system was decreased by 22%, compared to that of IFAS system. These findings are consistent with the results of Vitanza et al., and Saby et al. studies, which also reported excess sludge reduction efficiencies of 23.4%, and 36.8% in the OSA systems^25,29^. According to these findings, it can be concluded that modifying IFAS to an OSA system (IFAS-OSA) by adding an anoxic sludge holding tank (ASHT) in the return sludge line of the system with 4-h HRTs decreased the excess sludge production. Table6 shows the comparison of the operational parameters including ORP levels, sludge production coefficient (Y_obs_), and excess sludge reduction (Q_Excess_) of this study with Saby study.

### Filamentous *fungi* and yeasts in the IFAS and IFAS-OSA systems

As illustrated in Table 7 the total count of filamentous *fungi* and *yeasts* in different sites of the IFAS and IFAS-OSA systems were 1045 and 1721 (CFU/ml). The highest count of *fungi* in the IFAS and IFAS-OSA systems belonged to the genus *Aspergillus sp.* 302 (28.9%), 448 (26), and *Penicillium sp.* 223 (21.34), 321 (18.65), respectively, While the highest count of *yeasts* in the IFAS and IFAS-OSA systems were 196 (18.76) and 391(22.71), respectively. Thus, they could be the most effective genera in the biological nutrient (N & P) removal from the wastewater. This somewhat agrees with the results of Greben et al. study in 2007, who also stated that two of the six hyphomycetes isolates used for biological nitrogen removal (BNR) from the wastewater was *Penicillium sp.* These *fungi* had the ability to remove a large amount of nitrate nitrogen from the wastewater. Furthermore, in another study by Akhtar et al. the results showed that from the 9 species of *fungi*, *Aspergillus sp.* was the most effective species in the removal of NH_4_^+^–N. Different studies have also shown that various *fungi* have the ability to oxidize the reduced form of nitrogen^3,13^.

**Table 7:** Distribution of the total number (CFU/ml) and percentage frequency (%F) of various fungal genera and species in the IFAS and IFAS-OSA systems.

No	Genera of the *fungi*	Total N. (% F) IFAS	Total N. (% F) IFAS-OSA	Remarks*
1	*Aspergillus sp.*	302 (28.9)	448 (26)	Pur, Sap
2	*Penicillium sp.*	223 (21.34)	321 (18.65)	Pur, Sap
3	*Fusarium sp.*	52 (4.97)	75 (4.36)	Pur, Sap
4	*Cladosporium sp.*	40 (3.83)	56 (3.25)	Pur, Sap
5	*Alternaria sp.*	18 (1.72)	38 (2.2)	Pur, Sap
6	*Mucor sp.*	20 (1.91 )	33 (1.91)	Pur, Sap
7	*Rhizopus sp.*	24 (2.29)	45 (2.61)	Pur, Sap
8	*Stachybotrys sp.*	9 (0.86)	23 (1.33)	Pur, Sap
9	*Scopulariopsis sp.*	9 (0.86)	22 (1.27)	Pur, Sap
10	*Paecilomyces sp.*	15 (1.43)	22 (1.27)	Pur, Sap
11	*Aureobasidium sp.*	2 (0.19)	8 (0.46)	Pur, Sap
12	*chaetomium sp.*	2 (0.19)	12 (0.69)	Pur, Sap
13	*Yeasts*	196 (18.76)	391(22.71)	Pur, Sap
14	*Trichosporon sp.*	11 (1.05)	25 (1.45)	Pur, Sap
15	*Geotrichum sp.*	51 (4.88)	69 (4.12)	Pur, Sap
16	*Candida sp.*	40 (3.83)	82 (4.76)	Ind, Pur
17	*Rhodotorula sp.*	31 (2.96)	51 (2.96)	Ind, Pur
18	Total N of G	1045 (100)	1721(100)	-

Sites of sampling: I- anoxic tank, II- aeration tank, III- clarifier tank, IV- anoxic sludge holding tank (ASHT), V- effluent tank. N: Number, G: Genera, S: Species.

*Pur: Purifying; Sap: Saprophytic; Ind: Indicatory.

Due to the important role of the *Candida* species as an indicator in the purification process, the PCR result of the *Candida* species is demonstrated. Using classical and molecular identification techniques, 36 (90%) isolates of *C. albicans*, 3 (7.5%) isolates of *C. glabrata*, and one (2.5%) isolate of *C. kefyr* were identified in the IFAS system, and 73 (89%) isolates of *C. albicans*, 6 (7%) isolates of *C. glabrata*, and 3 (4%) isolate of *C. kefyr* were identified in the IFAS-OSA system.

### Protozoa and metazoa species in the IFAS and IFAS-OSA systems

**Figure 5:**
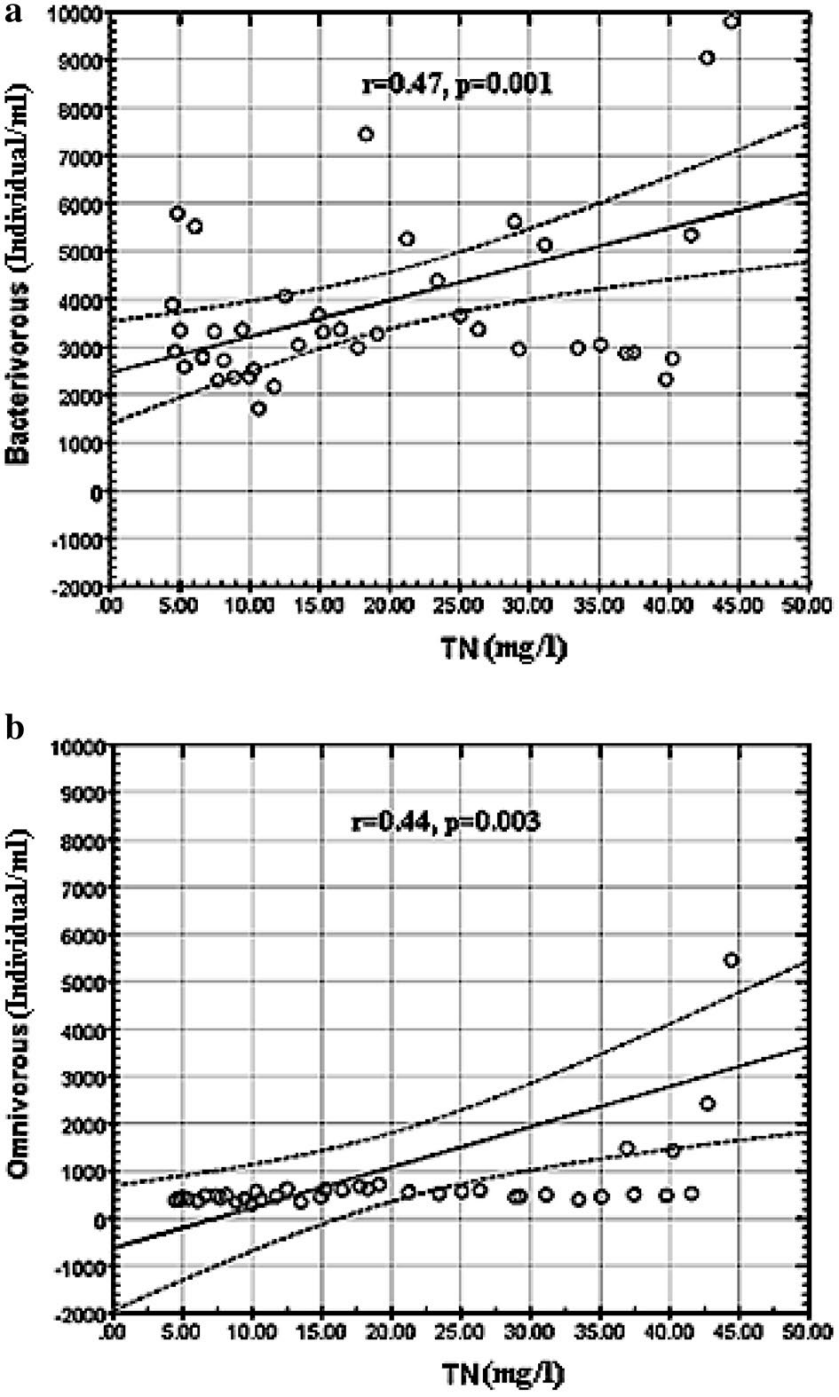
(**a**, **b**) Correlation between the trophic groups of protists and TN concentrations.

*Protozoa* and *metazoa* quantities of the IFAS and IFAS-OSA systems in different ecological groups changed considerably from April to June. According to Table 8 the highest average number (Indiv/Cm^3^) of *protozoa* species detected in the IFAS and IFAS-OSA systems were: *Euglypha acanthophora* (427, 469)*, Vorticella convallaria* (410, 476), *Aspidisca crenata* (350, 383)*, Vorticella infusionum* (305, 343)*, Acineria uncinata* (307, 328), *Euplotes Charon* (297, 354), *Arcella vulgaris* (252, 284), *Colpidium campylum* (195, 228), *Pyxidicula operculata* (194, 229), *Acineria incurvata (*181, 213). Although some studies have shown a high level of correlation between nitrogen removal efficiency and protists, such correlation has not been found yet for all kinds of *protozoa* and *metazoa* species^24^. In this research, there was no correlation between the ecological groups and total nitrogen (TN) concentration in the IFAS and IFAS-OSA bioreactors. However, a low level of correlation between total nitrogen (TN) concentration and the trophic groups of protists was detected. Thus omnivorous and bacterivorous numbers showed a weak positive correlation with total nitrogen (Fig. 5a, b). The current study also showed that there is no correlation between the variations in the concentration of nitrogen in the IFAS and IFAS-OSA bioreactors and the changes in the quantity of most recovered *protozoa* and *metazoa* species. According to the quantitative analyses, the activated sludge of the IFAS and upgraded IFAS-OSA bioreactors belonged to the first quality class in the Sludge Biotic Index (SBI) and is described as stable and well-colonized with a high biological activity^21^.

**Table 8:** Distribution of the *protozoa* and *metazoa* genera and species (Indiv/Cm^3^) in the IFAS and IFAS-OSA systems.

*Protozoa* and *metazoa* genera and species	M1-M3 IFAS				M1-M3 IFAS-OSA				Trophic group	Ecological group
	Mean	SD	Max	Min	Mean	SD	Max	Min		
***Ciliate sp.***										
*Aspidisca crenata* (Domergue, 1885)	350	123	1248	25	383	139	1542	96	Ba	Cr
*Aspidisca costata* (Dujardin, 1841)	157	21	376	42	155	11	365	24	Ba	Cr
*Aspidisca lynceus* (Muller, 1773)	132	7	300	20	135	8	242	26	Ba	Cr
*Aspidisca cicada* (Muller, 1786)	158	7	404	40	165	11	376	54	Ba	Cr
*Acineria incurvata* (Dujardin, 1841)	181	67	654	56	213	94	621	43	Ba	Cr
*Acineria uncinata* (Tucolesco, 1962)	307	210	888	41	328	221	921	87	Ba	Cr
*Euplotes Charon* (Muller, 1786)	297	269	1454	33	354	285	1654	86	Ba, Fl	Cr
*Blepharisma sp.* (Perty, 1852)	113	95	346	22	130	100	286	67	Ba, Fl	Sw
*Colpidium colpoda* (Losana, 1829)	87	8	144	38	118	27	143	89	Ba	Sw
*Colpidium campylum* (Stokes,1886)	195	37	326	24	228	43	275	98	Ba	Sw
*Vorticella infusionum* (Dujardin, 1841)	305	270	1570	6	343	290	1678	155	Ba	A
*Vorticella microstoma* (Ehrenberg, 1830)	150	55	212	21	171	62	201	17	Ba	A
*Vorticella convallaria* (Linnaeus, 1767)	410	433	1768	32	476	582	1846	85	Ba	A
*Paramecium aurelia* (Ehrhart, 1742)	76	10	128	22	89	12	102	8	Om	Sw
*Paramecium caudatum,* (Thunberg, 1743)	82	26	224	22	124	47	167	7	Om	Sw
***Testate amoebae***										
*Euglypha acanthophora* (Ehrenberg, 1838)	427	324	1269	24	469	357	1365	68	Ba	Cr
*Arcella vulgaris* (Ehrenberg, 1832)	252	57	674	22	284	62	654	34	Ba	Cr
*Pyxidicula operculata* (Ehrenberg, 1838)	194	46	615	80	229	52	688	88	Ba	Cr
*Naked amoebae* (Saintvincent,1822)	29	16	54	4	58	17	47	3	Ba	Cr
***Rotifer sp.***										
*Lecan clara* (Bryce, 1892)	52	9	108	18	71	16	85	5	Om	Sw
*Lecan agilis* (Bryce, 1892)	51	16	90	5	60	13	70	4	Om	Sw
*Lecane inquieta* (Myers, 1936)	48	3	78	20	50	15	65	3	Om	Sw
*Brachionus plicatilis* (Muller, 1786)	54	10	108	25	51	16	66	4	Om	Sw
*Macrotrachela habita* (Bryce, 1894)	46	3	84	22	42	10	52	3	Om	Sw
*Macrotrachela plicata* (Bryce,1892)	32	12	75	18	40	14	57	5	Om	Sw
*Nematode sp.* (Diesing, 1861)	44	1	70	5	38	16	56	7	Om	Sw

Trophic groups: Ba-bacterivorous; F-consumer of heterotrophic flagellates; Om – omnivorous; Ecological groups: A- attached; Cr- crawling; Sw- free swimming; M1: First month (April); M2: Second month (May); M3: Third month (June), Indiv: Individual.

**Mean, Min, and Max: the average, minimum, and maximum (Indiv/Cm^3^) of the *protozoa* and *metazoa* genera and species from different sites of the IFAS and the IFAS-OAS systems.

Additionally the respirometric analysis was conducted on dedicated device (BioCargo laboratory respirometer, GIG, EMAG SERWIS, Poland) in order to show proper physiological state of activated sludge and its high biological activity. The control of respiratory activity of the activated sludge and the determination for OUR sludge value in the endogenous state, showed no negative correlation between TN and activated sludge analysis. Such approach using SBI and respirometric analysis allows to obtain full scope of biological system operation^12^.

#### Total cost and energy analyses

Table 9 shows the comparison of total cost and energy analyses (per month and per year) of the present work with other literature. AS illustrated in Table 9 total Cost of IFAS-OSA system per year was decreased by 21.98% compared with that of the IFAS system. Total Cost of IFAS-OSA system per year was decreased by 35.63% compared with that of the MLE-OSA system.

**Table 9:** Comparison of cost and energy analyses of the present study with other literature.

Cost and energy analyses	Total cost(R)	Total cost(R)	Study
	Per month	Per year	
IFAS	847,900,000	10,133,816,169	This study
IFAS-OSA	656,360,000	7,906,910,111	
MLE	1،830،800،000	21،969،600،000	Nikpour et al.^22^
MLE-OSA	1،023،600،000	12،283،200،000	

## Conclusions

The new upgraded IFAS-OSA system with SRT of 10 days and HRT of 4 h in the ASHT, demonstrated the better performance in the removal of total nitrogen, phosphorus, COD, and reduction of excess sludge compared to that of the control system. The average TN removal efficiencies were 80 ± 2.5%, 86 ± 1.7%, while the average PO4-P removal efficiencies were 33 ± 8.2% and 42 ± 3.6% in the IFAS and IFAS-OSA systems. COD removal efficiencies were 92 ± 0.65%, 97 ± 0.52%, respectively. Biomass yield coefficient (Y_obs_) in the IFAS and IFAS-OSA systems were 0.44 and 0.24 (gr MLSS/ gr COD). Hence, sludge production decreased by 45%.The new upgraded IFAS-OSA system also showed a better performance for sludge settleability. According to the findings, this research provided the new foundation for future studies on specific species of protists, *fungi* and their effects on biological nutrient removal processes.

## Data Availability

Please contact author for data requests.

## Abbreviations

ASHT: Anoxic sludge holding tank
BNR: Biological nutrient removal
BOD: Biological oxygen demand
CAS: Conventional activated sludge
CFU: Colony forming unit
COD: Chemical oxygen demand
DO: Dissolved oxygen
ESR: Excess sludge reduction
HRT: Hydraulic retention time
IFAS: Integrated fixed-film activated sludge
IFAS-OSA: Integrated fixed film activated sludge-oxic settling anoxic
ITS: Internal spacer region
MLE: Modified Ludzack-Ettinger
MLSS: Mixed liquor suspended solids
NH4^+^-N: Ammonium nitrogen
NO_3_^–^N: Nitrate nitrogen
ORP: Oxidation–reduction potential
OLR: Organic loading rate
OSA: Oxic-settling-anoxic
OUR: Oxygen uptake rate
PCR–RFLP: Polymerase chain reaction-restriction fragment length polymorphism
PAO: Phosphorus accumulating organisms
PO4-3-P: Orthophosphate-phosphorus
Q _Excess_: Excess sludge flow rate
RAS: Return activated sludge
SBI: Sludge biotic index
SDA: Sabouraud dextrose agar
SRT: Solid retention time
SVI: Sludge volume index
TN: Total nitrogen
TSS: Total suspended solids
TKN: Total Kjeldahl nitrogen
Y_obs_: Observed yield coefficient
